# Supplementary material for: Can individual subjective confidence in training questions predict group performance in test questions?
Source: PLoS One. 2023 Mar 7;18(3):e0280984. doi: 10.1371/journal.pone.0280984 (PMC9990919; doi:10.1371/journal.pone.0280984)
Supplement: S1 File — (DOCX) [file pone.0280984.s001.docx]

**Supporting Information**

Can individual subjective confidence in training questions predict group performance in test questions?

## All data, code, and materials used in our experiments are available at

<https://osf.io/rj4dv/>

**Supporting Information 1**

## Procedure for selecting questions in a *population inference task*.

We selected 70 of the 210 questions (i.e., all possible 105 pairs using 15 Japanese cities in the “difficult list” + all possible 105 pairs using 15 Japanese cities in the “easy list”) from the original study [1]. We adhered to the following procedure:

1. For each difficulty list (“difficult list” or “easy list”), we sorted all questions according to the question number in the original study and provided Question ID for the current study (easy and difficult questions were assigned to 1~105 and 106~210, respectively).
2. To ensure equal representation of both difficult and easy questions, we picked up every three questions (i.e., Question ID 1, 4, 7, …, 205, 208).

## Question list (population inference task: 70 questions)

Column names legend:

altCorrect: the correct alternative (i.e., larger city) in the Japanese description.

city_large_En: the correct alternative (i.e., larger city) in the English description.

city_large_popu: population in larger city.

altFalse: the false alternative (i.e., smaller city) in the Japanese description.

city_small_En: the false alternative (i.e., smaller city) in the English description.

city_small_popu: population in smaller city.

difficulty: difficulty levels in the original study (i.e., difficult or easy lists).

qidx: Question ID.

| altCorrect | city_large_En | city_large_popu | altFalse | city_small_En | city_small_popu | difficulty | qidx |
| --- | --- | --- | --- | --- | --- | --- | --- |
| 横浜市 | Yokohama-shi | 3724844 | 大阪市 | Osaka-shi | 2691185 | easy | 1 |
| 横浜市 | Yokohama-shi | 3724844 | 福岡市 | Fukuoka-shi | 1538681 | easy | 4 |
| 横浜市 | Yokohama-shi | 3724844 | 広島市 | Hiroshima-shi | 1194034 | easy | 7 |
| 横浜市 | Yokohama-shi | 3724844 | 新潟市 | Niigata-shi | 810157 | easy | 10 |
| 横浜市 | Yokohama-shi | 3724844 | 岡山市 | Okayama-shi | 719474 | easy | 13 |
| 大阪市 | Osaka-shi | 2691185 | 札幌市 | Sapporo-shi | 1952356 | easy | 16 |
| 大阪市 | Osaka-shi | 2691185 | 京都市 | Kyoto-shi | 1475183 | easy | 19 |
| 大阪市 | Osaka-shi | 2691185 | 千葉市 | Chiba-shi | 971882 | easy | 22 |
| 大阪市 | Osaka-shi | 2691185 | 熊本市 | Kumamoto-shi | 740822 | easy | 25 |
| 名古屋市 | Nagoya-shi | 2295638 | 札幌市 | Sapporo-shi | 1952356 | easy | 28 |
| 名古屋市 | Nagoya-shi | 2295638 | 京都市 | Kyoto-shi | 1475183 | easy | 31 |
| 名古屋市 | Nagoya-shi | 2295638 | 千葉市 | Chiba-shi | 971882 | easy | 34 |
| 名古屋市 | Nagoya-shi | 2295638 | 熊本市 | Kumamoto-shi | 740822 | easy | 37 |
| 札幌市 | Sapporo-shi | 1952356 | 福岡市 | Fukuoka-shi | 1538681 | easy | 40 |
| 札幌市 | Sapporo-shi | 1952356 | 広島市 | Hiroshima-shi | 1194034 | easy | 43 |
| 札幌市 | Sapporo-shi | 1952356 | 新潟市 | Niigata-shi | 810157 | easy | 46 |
| 札幌市 | Sapporo-shi | 1952356 | 岡山市 | Okayama-shi | 719474 | easy | 49 |
| 福岡市 | Fukuoka-shi | 1538681 | 京都市 | Kyoto-shi | 1475183 | easy | 52 |
| 福岡市 | Fukuoka-shi | 1538681 | 千葉市 | Chiba-shi | 971882 | easy | 55 |
| 福岡市 | Fukuoka-shi | 1538681 | 熊本市 | Kumamoto-shi | 740822 | easy | 58 |
| 神戸市 | Kobe-shi | 1537272 | 京都市 | Kyoto-shi | 1475183 | easy | 61 |
| 神戸市 | Kobe-shi | 1537272 | 千葉市 | Chiba-shi | 971882 | easy | 64 |
| 神戸市 | Kobe-shi | 1537272 | 熊本市 | Kumamoto-shi | 740822 | easy | 67 |
| 京都市 | Kyoto-shi | 1475183 | 広島市 | Hiroshima-shi | 1194034 | easy | 70 |
| 京都市 | Kyoto-shi | 1475183 | 新潟市 | Niigata-shi | 810157 | easy | 73 |
| 京都市 | Kyoto-shi | 1475183 | 岡山市 | Okayama-shi | 719474 | easy | 76 |
| 広島市 | Hiroshima-shi | 1194034 | 千葉市 | Chiba-shi | 971882 | easy | 79 |
| 広島市 | Hiroshima-shi | 1194034 | 熊本市 | Kumamoto-shi | 740822 | easy | 82 |
| 仙台市 | Sendai-shi | 1082159 | 千葉市 | Chiba-shi | 971882 | easy | 85 |
| 仙台市 | Sendai-shi | 1082159 | 熊本市 | Kumamoto-shi | 740822 | easy | 88 |
| 千葉市 | Chiba-shi | 971882 | 新潟市 | Niigata-shi | 810157 | easy | 91 |
| 千葉市 | Chiba-shi | 971882 | 岡山市 | Okayama-shi | 719474 | easy | 94 |
| 新潟市 | Niigata-shi | 810157 | 熊本市 | Kumamoto-shi | 740822 | easy | 97 |
| 浜松市 | Hamamatsu-shi | 797980 | 熊本市 | Kumamoto-shi | 740822 | easy | 100 |
| 熊本市 | Kumamoto-shi | 740822 | 岡山市 | Okayama-shi | 719474 | easy | 103 |
| 川口市 | Kawaguchi-shi | 578112 | 町田市 | Machida-shi | 432348 | difficult | 106 |
| 川口市 | Kawaguchi-shi | 578112 | 郡山市 | Koriyama-shi | 335444 | difficult | 109 |
| 川口市 | Kawaguchi-shi | 578112 | 八戸市 | Hachinohe-shi | 231257 | difficult | 112 |
| 川口市 | Kawaguchi-shi | 578112 | 高岡市 | Takaoka-shi | 172125 | difficult | 115 |
| 川口市 | Kawaguchi-shi | 578112 | 今治市 | Imabari-shi | 158114 | difficult | 118 |
| 町田市 | Machida-shi | 432348 | 津市 | Tsu-shi | 340973 | difficult | 121 |
| 町田市 | Machida-shi | 432348 | 松本市 | Matsumoto-shi | 243293 | difficult | 124 |
| 町田市 | Machida-shi | 432348 | 日立市 | Hitachi-shi | 185054 | difficult | 127 |
| 町田市 | Machida-shi | 432348 | 大垣市 | Ogaki-shi | 159879 | difficult | 130 |
| 高崎市 | Takasaki-shi | 370884 | 津市 | Tsu-shi | 340973 | difficult | 133 |
| 高崎市 | Takasaki-shi | 370884 | 松本市 | Matsumoto-shi | 243293 | difficult | 136 |
| 高崎市 | Takasaki-shi | 370884 | 日立市 | Hitachi-shi | 185054 | difficult | 139 |
| 高崎市 | Takasaki-shi | 370884 | 大垣市 | Ogaki-shi | 159879 | difficult | 142 |
| 津市 | Tsu-shi | 340973 | 郡山市 | Koriyama-shi | 335444 | difficult | 145 |
| 津市 | Tsu-shi | 340973 | 八戸市 | Hachinohe-shi | 231257 | difficult | 148 |
| 津市 | Tsu-shi | 340973 | 高岡市 | Takaoka-shi | 172125 | difficult | 151 |
| 津市 | Tsu-shi | 340973 | 今治市 | Imabari-shi | 158114 | difficult | 154 |
| 郡山市 | Koriyama-shi | 335444 | 松本市 | Matsumoto-shi | 243293 | difficult | 157 |
| 郡山市 | Koriyama-shi | 335444 | 日立市 | Hitachi-shi | 185054 | difficult | 160 |
| 郡山市 | Koriyama-shi | 335444 | 大垣市 | Ogaki-shi | 159879 | difficult | 163 |
| 佐世保市 | Sasebo-shi | 255439 | 松本市 | Matsumoto-shi | 243293 | difficult | 166 |
| 佐世保市 | Sasebo-shi | 255439 | 日立市 | Hitachi-shi | 185054 | difficult | 169 |
| 佐世保市 | Sasebo-shi | 255439 | 大垣市 | Ogaki-shi | 159879 | difficult | 172 |
| 松本市 | Matsumoto-shi | 243293 | 八戸市 | Hachinohe-shi | 231257 | difficult | 175 |
| 松本市 | Matsumoto-shi | 243293 | 高岡市 | Takaoka-shi | 172125 | difficult | 178 |
| 松本市 | Matsumoto-shi | 243293 | 今治市 | Imabari-shi | 158114 | difficult | 181 |
| 八戸市 | Hachinohe-shi | 231257 | 日立市 | Hitachi-shi | 185054 | difficult | 184 |
| 八戸市 | Hachinohe-shi | 231257 | 大垣市 | Ogaki-shi | 159879 | difficult | 187 |
| 山口市 | Yamaguchi-shi | 197422 | 日立市 | Hitachi-shi | 185054 | difficult | 190 |
| 山口市 | Yamaguchi-shi | 197422 | 大垣市 | Ogaki-shi | 159879 | difficult | 193 |
| 日立市 | Hitachi-shi | 185054 | 高岡市 | Takaoka-shi | 172125 | difficult | 196 |
| 日立市 | Hitachi-shi | 185054 | 今治市 | Imabari-shi | 158114 | difficult | 199 |
| 高岡市 | Takaoka-shi | 172125 | 大垣市 | Ogaki-shi | 159879 | difficult | 202 |
| 都城市 | Miyakonojo-shi | 165029 | 大垣市 | Ogaki-shi | 159879 | difficult | 205 |
| 大垣市 | Ogaki-shi | 159879 | 今治市 | Imabari-shi | 158114 | difficult | 208 |

**Supporting Information 2**

## Procedure for selecting questions in a *relationships comparison task*.

We selected 25 of the 100 questions from Study 2b in the original study [2] based on the rates of correct judgments using the following procedure:

1. To avoid ceiling effects, we accepted questions with a mean accuracy (i.e., rates of correct judgments) lower .90 in the original study. As a result, we gathered 50 questions (50 city names and 21 country names, and therefore 71 objects in total).
2. We sorted these 50 questions in the order of the mean of accuracy.
3. To ensure equal representation of both difficult and easy questions, we selected every two questions (i.e., rank of the mean accuracy 1^st^, 3^rd^, …, 49^th^).

## Question list (relationships comparison task: 25 questions)

Column names legend:

city: city name presented in a question sentence in the Japanese description.

city_ En: city name in the English description.

altCorrect: the correct alternative (i.e., country in which the presented city is) in the Japanese description.

altCorrect_En: the correct alternative in the English description.

altFalse: the false alternative (i.e., country in which the presented city is NOT) in the Japanese description.

altFalse_En: the false alternative in the English description.

meanCorrectRate: the mean accuracy in the question reported in the original study.

qidx: Question ID.

| city | city_En | altCorrect | altCorrect_En | altFalse | altFalse_En | meanCorrectRate | qidx |
| --- | --- | --- | --- | --- | --- | --- | --- |
| ブラジリア | Brasilia | ブラジル | Brazil | ナイジェリア | Nigeria | 0.86275 | 1 |
| アジスアベバ | Addis Ababa | エチオピア | Ethiopia | エジプト | Egypt | 0.82353 | 2 |
| アダマ | Adaamaa | エチオピア | Ethiopia | イラン | Iran | 0.60784 | 3 |
| アレクサンドリア | Alexandria | エジプト | Egypt | コンゴ民主共和国 | Democratic Rep. of the Congo | 0.88235 | 4 |
| セビリア | Seville | スペイン | Spain | ウクライナ | Ukraine | 0.37255 | 5 |
| オデーサ | Odessa | ウクライナ | Ukraine | スーダン | Sudan | 0.54902 | 6 |
| アルハルツームバフリ | al-Khartûm Bahrî | スーダン | Sudan | ウガンダ | Uganda | 0.58824 | 7 |
| ニャラ | Nyala | スーダン | Sudan | イラク | Iraq | 0.70588 | 8 |
| アルビル | Erbil | イラク | Iraq | サウジアラビア | Saudi Arabia | 0.4902 | 9 |
| フェズ | Fez | モロッコ | Morocco | サウジアラビア | Saudi Arabia | 0.72549 | 10 |
| バルキシメト | Barquisimeto | ベネズエラ | Venezuela | ウズベキスタン | Uzbekistan | 0.39216 | 11 |
| グアヤナ | Guayana | ベネズエラ | Venezuela | モザンビーク | Mozambique | 0.47059 | 12 |
| サマルカンド | Samarkand | ウズベキスタン | Uzbekistan | モザンビーク | Mozambique | 0.64706 | 13 |
| ナンプラ | Nampula | モザンビーク | Mozambique | イエメン | Yemen | 0.52941 | 14 |
| サルヴァドール | Salvador | ブラジル | Brazil | エチオピア | Ethiopia | 0.62745 | 15 |
| イバダン | Ibadan | ナイジェリア | Nigeria | エチオピア | Ethiopia | 0.56863 | 16 |
| ゴンダール | Gondar | エチオピア | Ethiopia | コンゴ民主共和国 | Democratic Rep. of the Congo | 0.27451 | 17 |
| メックエル | Mäqälle | エチオピア | Ethiopia | イギリス | British | 0.82353 | 18 |
| キャラジ | Karaj | イラン | Iran | ミャンマー | Myanmar | 0.5098 | 19 |
| マンダレー | Mandalay | ミャンマー | Myanmar | ウクライナ | Ukraine | 0.60784 | 20 |
| モーラミャイン | Mawlamyaing | ミャンマー | Myanmar | スーダン | Sudan | 0.68627 | 21 |
| ハルキウ | Kharkiv | ウクライナ | Ukraine | モロッコ | Morocco | 0.47059 | 22 |
| ハルツーム | Khartoum | スーダン | Sudan | モロッコ | Morocco | 0.54902 | 23 |
| カンパラ | Kampala | ウガンダ | Uganda | サウジアラビア | Saudi Arabia | 0.7451 | 24 |
| カラカス | Caracas | ベネズエラ | Venezuela | イエメン | Yemen | 0.41176 | 25 |

**Supporting Information 3**

## General tendencies of the behavioral data.

We confirmed the general tendencies of behavioral data. The x-axis and y-axis show the participant ID and question ID, respectively, and each cell denotes each participant’s judgment (correct: red cell; false: blue cell). Darker colors denote higher confidence ratings. For each question, 15 individuals in the highest 15 confidence ratings are surrounded by black lines (in some questions, there are more than 15 individuals because of a tie). As the figure shows, few participants consistently had high confidence in a task set (i.e., for many participants, the black-squared cells sparsely aligned on columns).


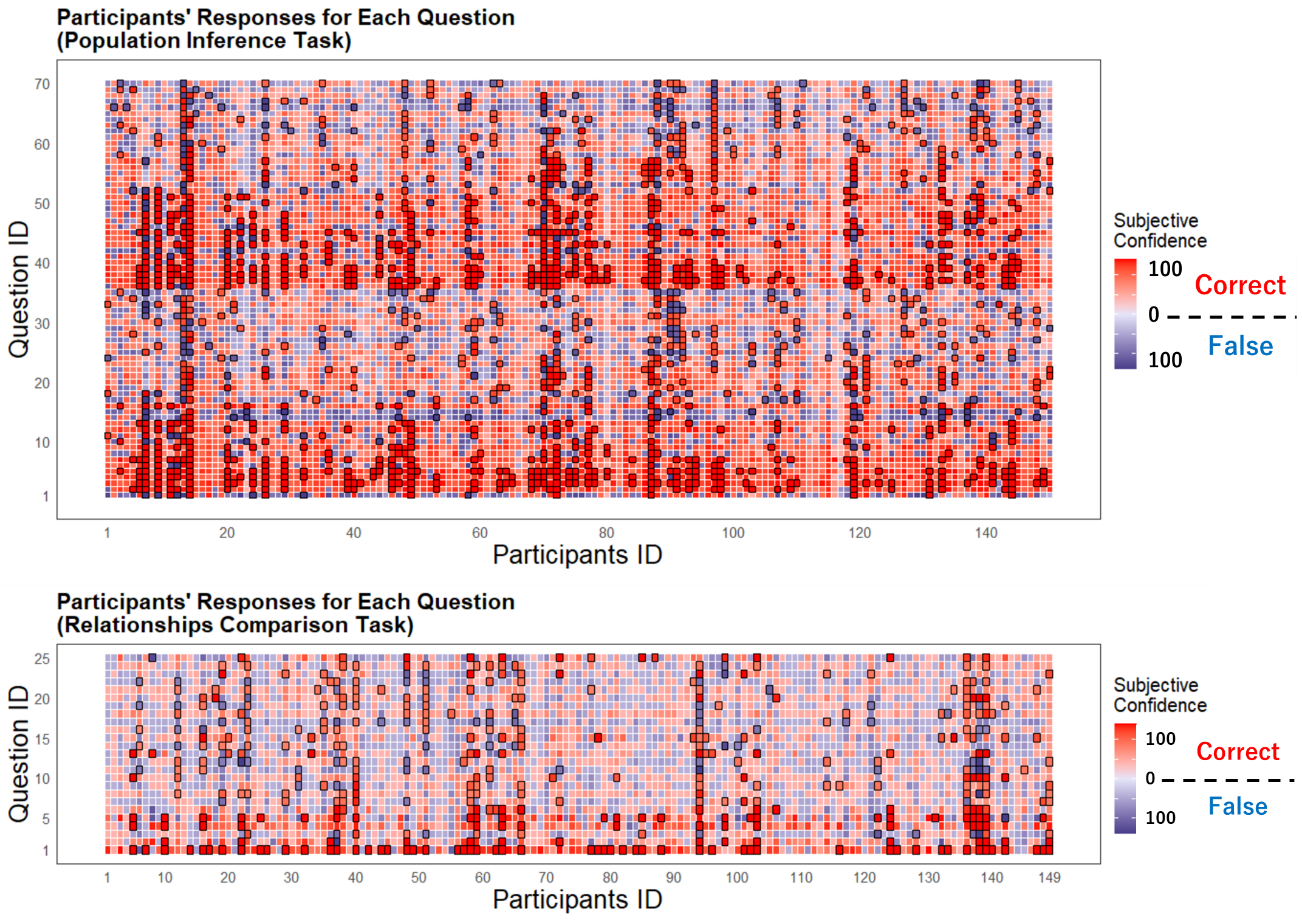


**Supporting Information 4**

## MCMC results: Convergence and distribution of the estimated parameter (confidence) for each question.

Using “brms,” an R package to conduct analyses by a generalized linear model (GLM) applying Markov Chain Monte Carlo (MCMC) simulations [3], we predicted correct/false responses from participants’ confidence ratings for each question. We set a non-informative prior as a prior distribution and ran MCMC by 3000 iterations, 1500 warmups, and 3 chains. Although most parameters converged successfully, a strange distribution and trace plot were observed in one question (the sixth from the top and second from the left for the “within-questions,” Question 37 of a population inference task. This was probably because all participants made correct judgments). We omitted the question from the GLM predictions.

### Population inference task (70 questions)

### Posterior distributions and trace plots of the coefficients of confidence


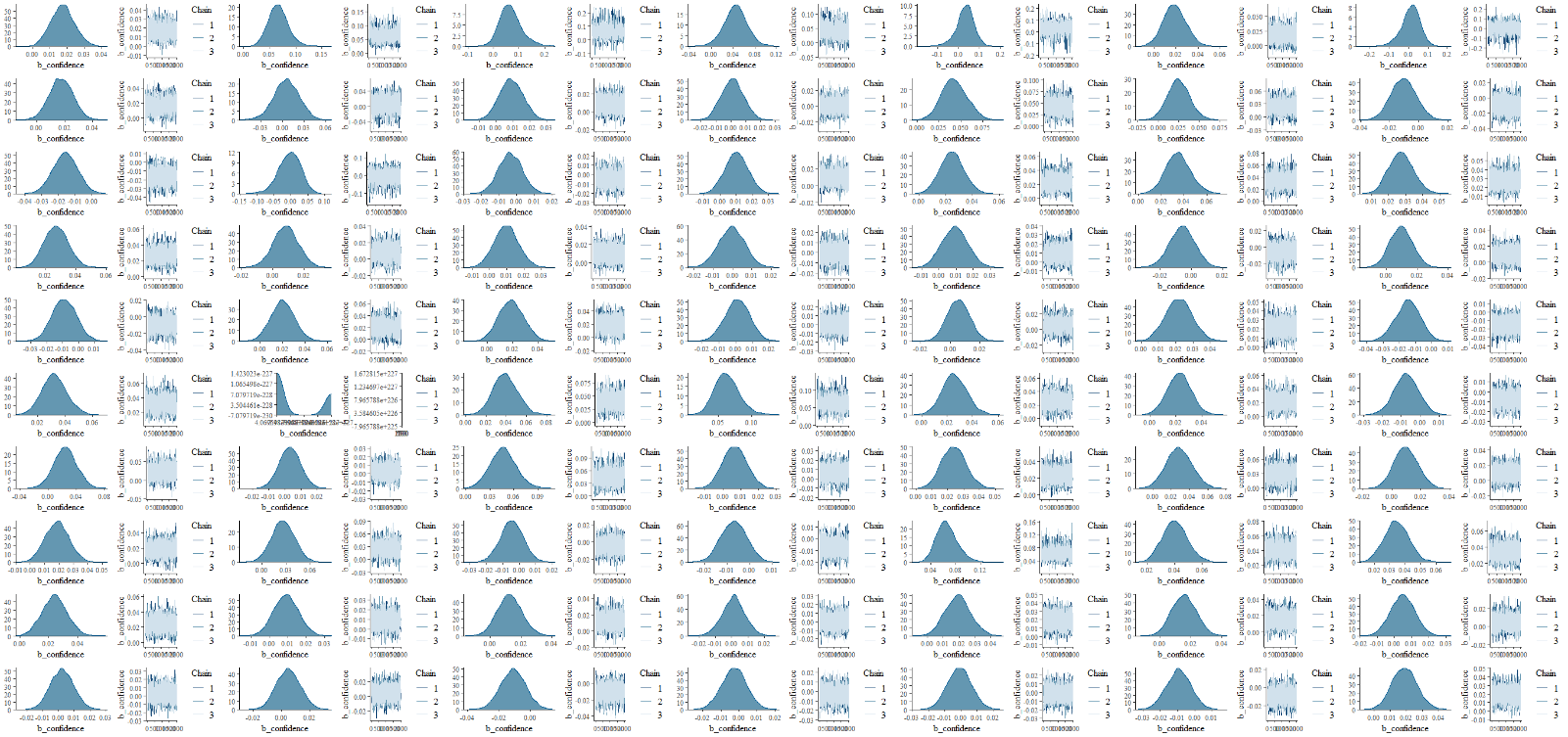


### Forest plots of coefficients of confidence


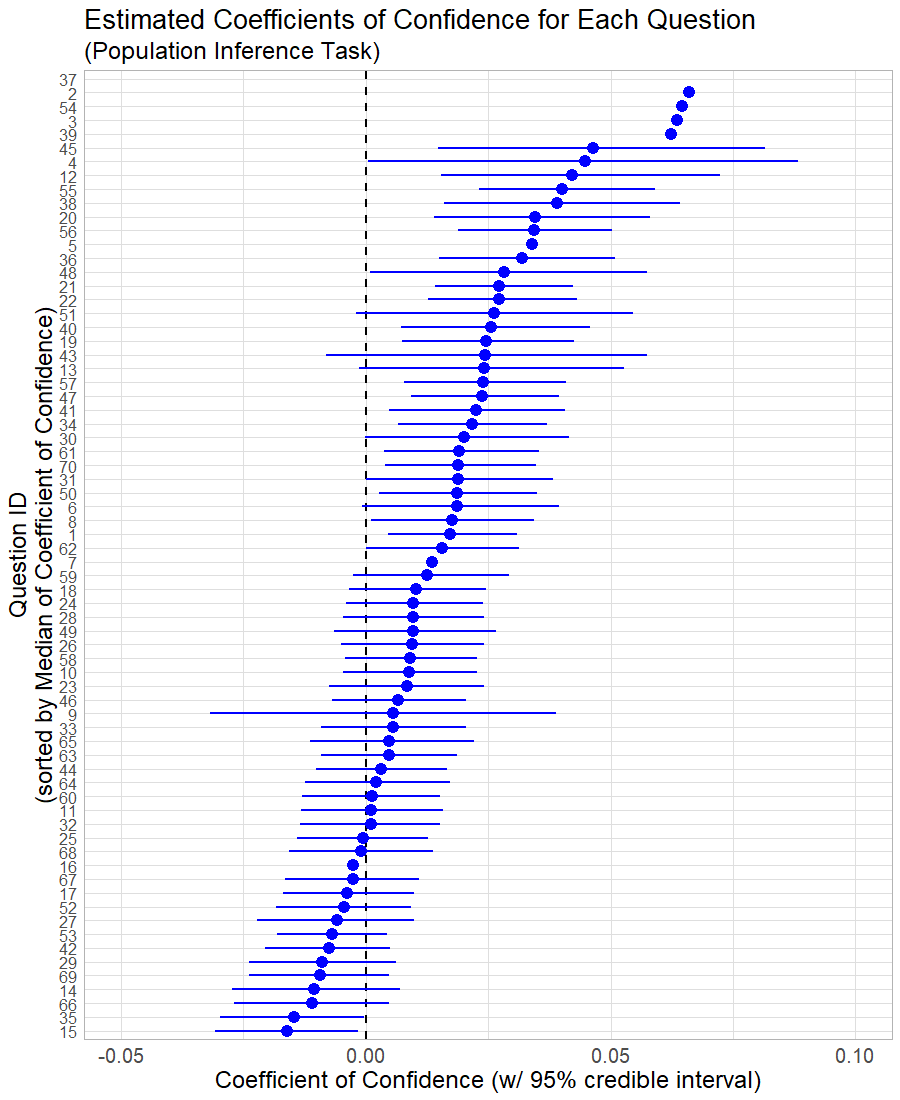


Note: Dots and error bars denote the median of the coefficients and 95% credible intervals (CI), respectively. If the 95% CI was outside the range of the x-axis (-0.05 ~ 0.10), its error bar was omitted.

### Relationships comparison task (25 questions)

### Posterior distributions and trace plots of the coefficients of confidence


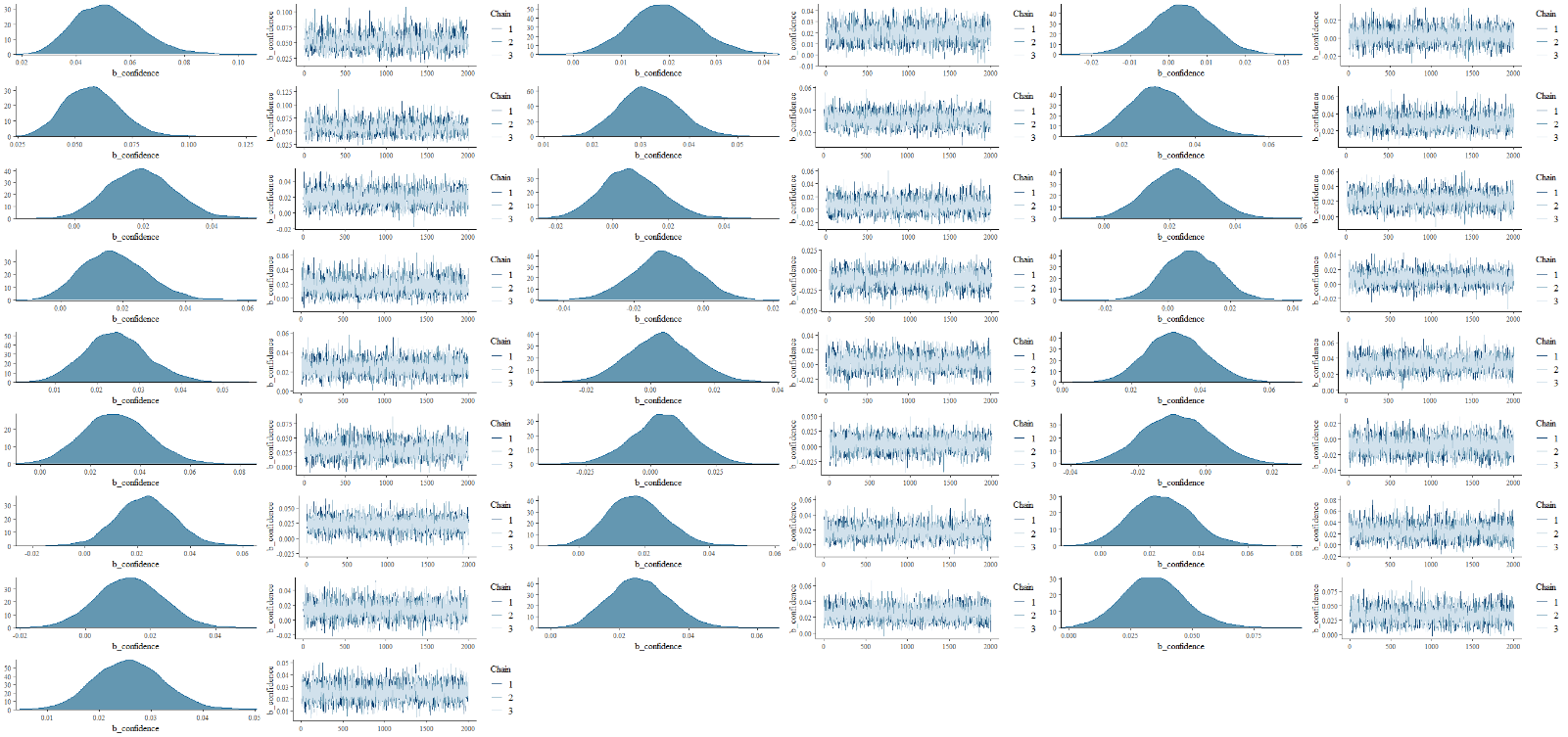


### Forest plots of coefficients of confidence


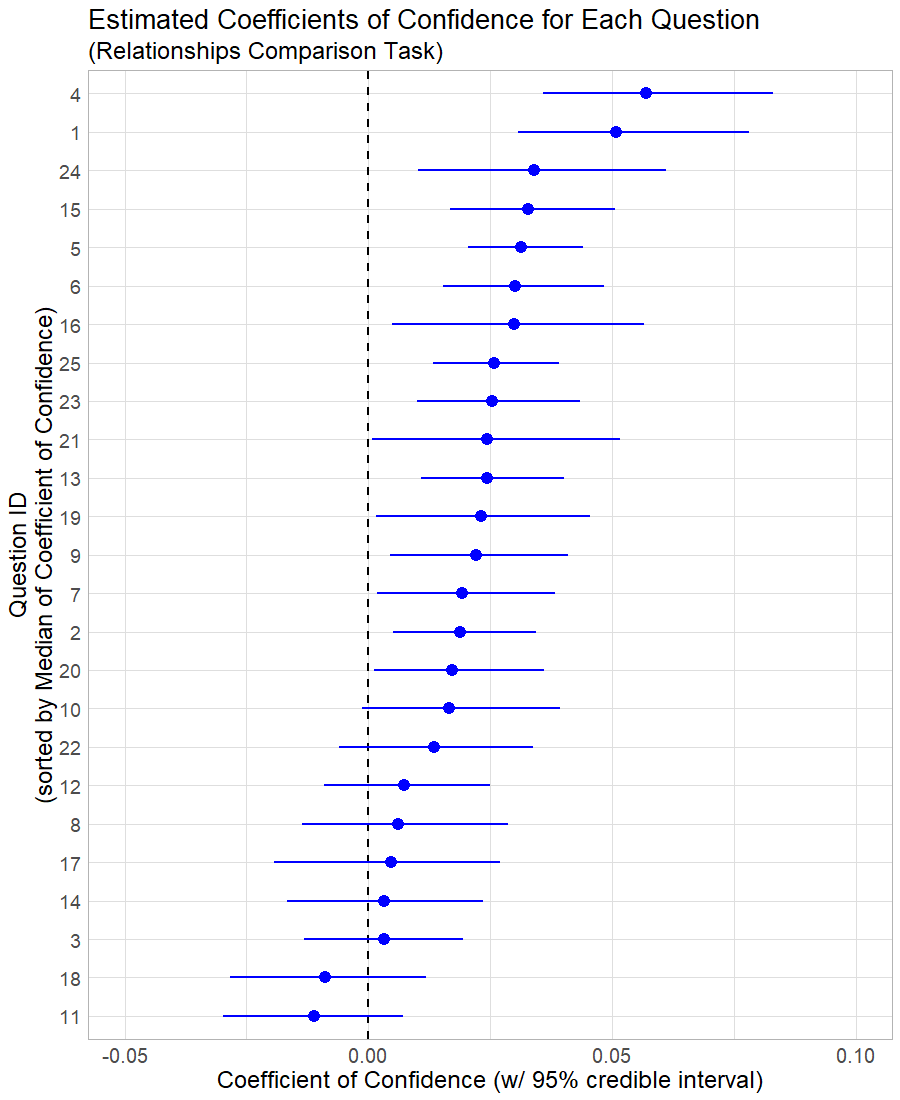


Note: Dots and error bars denote the median of the coefficients and 95% credible intervals (CI), respectively.

**Supporting Information 5**

## Differences between accuracy in training questions and test questions provided in the computer simulations (results for all conditions).

Each group’s difference between test accuracy and training accuracy is shown by the bars, and the proportions of the “test worse” cases (i.e., test accuracy – training accuracy < 0) and the mean differences in test worse cases are summarized in the lower tables.


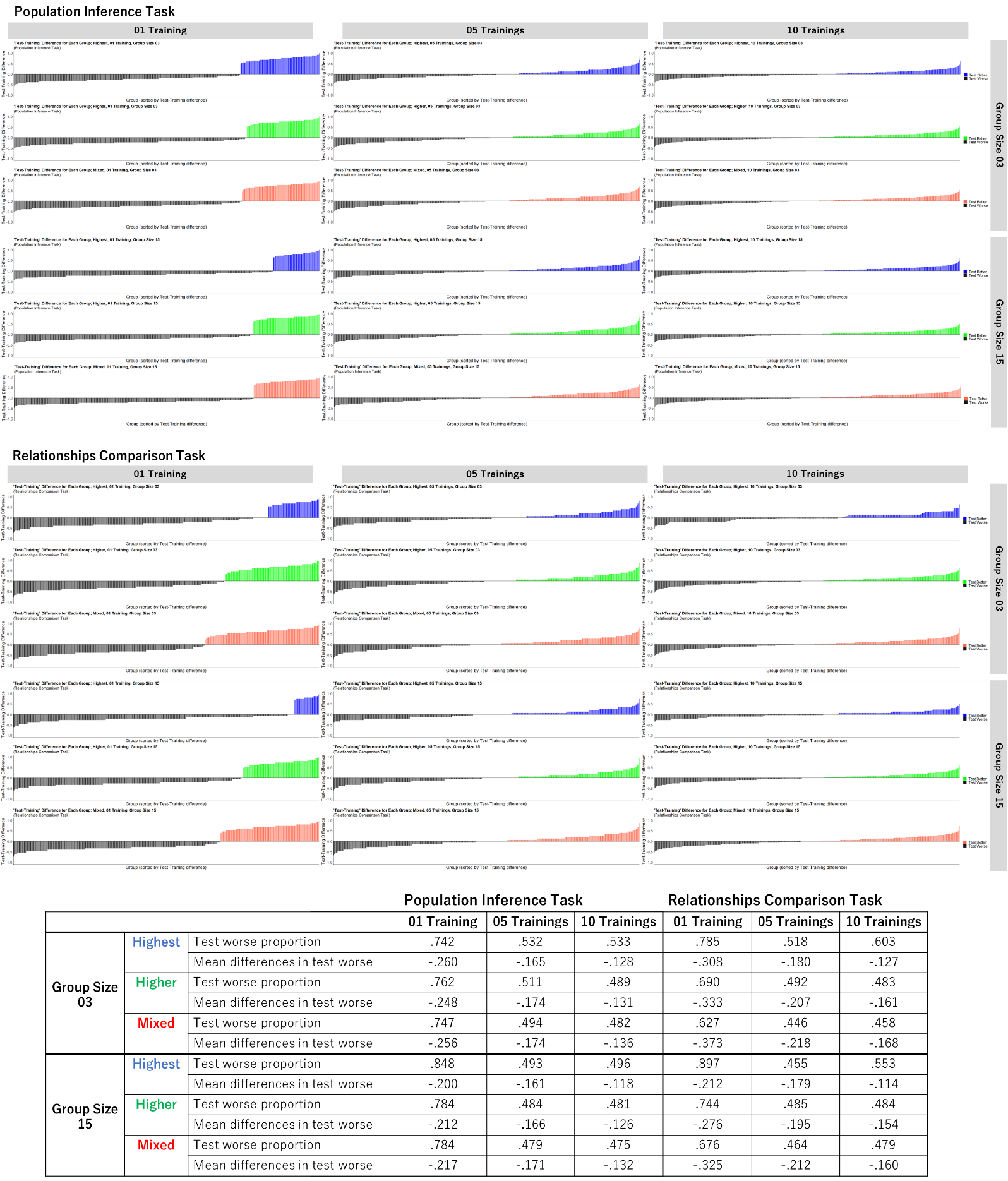


**Supporting Information 6**

## Differences between accuracy in training questions and test questions provided in the computer simulations (shown in the form of heatmaps).

We counted how often the patterns of accuracy (e.g., 2 patterns in 1 training question, 0% or 100%; 26 patterns in 25 test questions, 0%, 4%, 8%, …, 96%, or 100%) were observed in 5000 iterations. The depth of the colors and values in the tiles denote the frequency observed over 5000 iterations of the computer simulations. The x-axis and y-axis show the accuracy of the training and test questions, respectively. Gray zones (tiles surrounded by black lines) show the “test-worse” cases (i.e., test accuracy – training accuracy < 0). The depth of colors and values in the tiles denote the frequency observed in 5000 iterations of the computer simulations.

## Population inference task (70 questions); group size 3


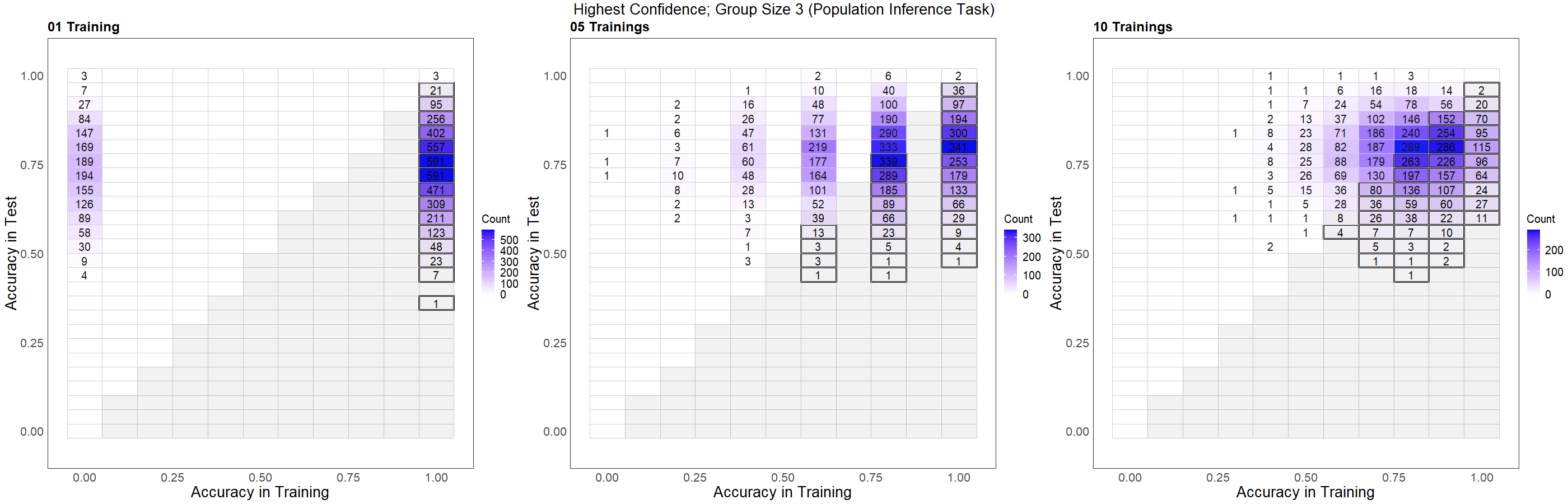


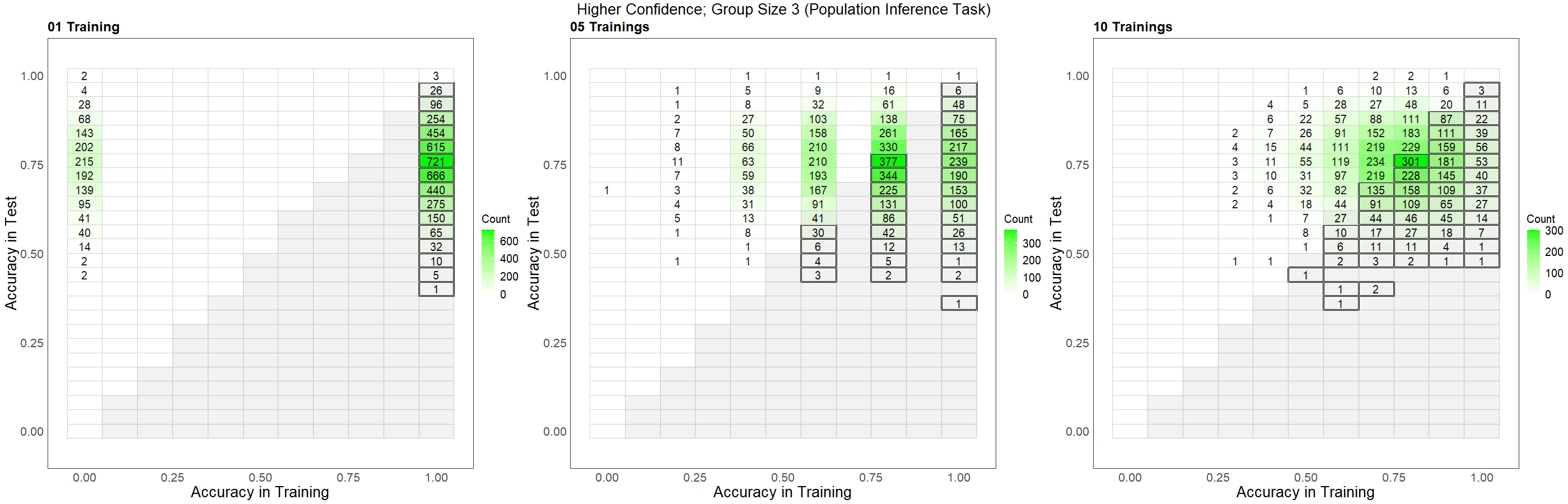


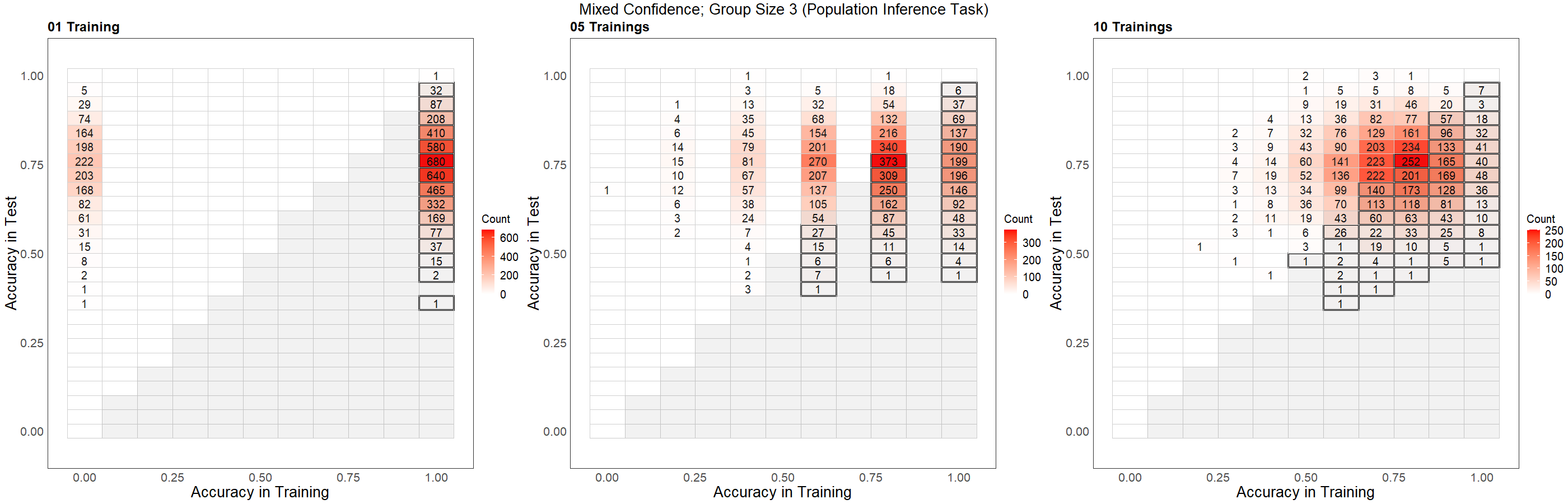


## Population inference task (70 questions); group size 15


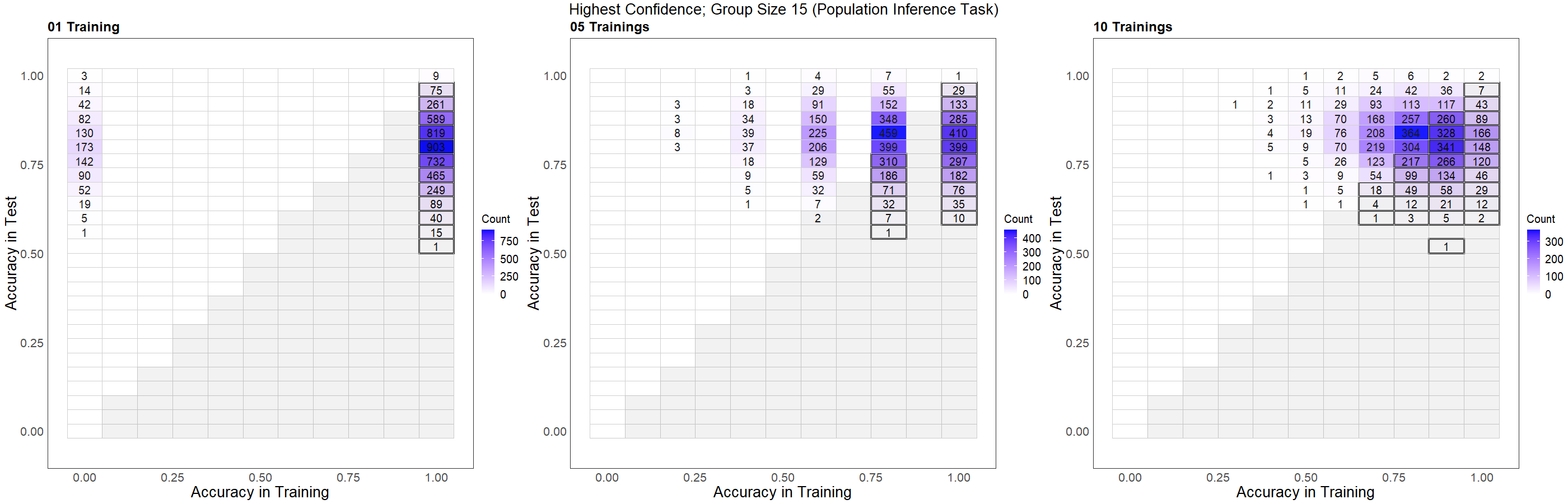


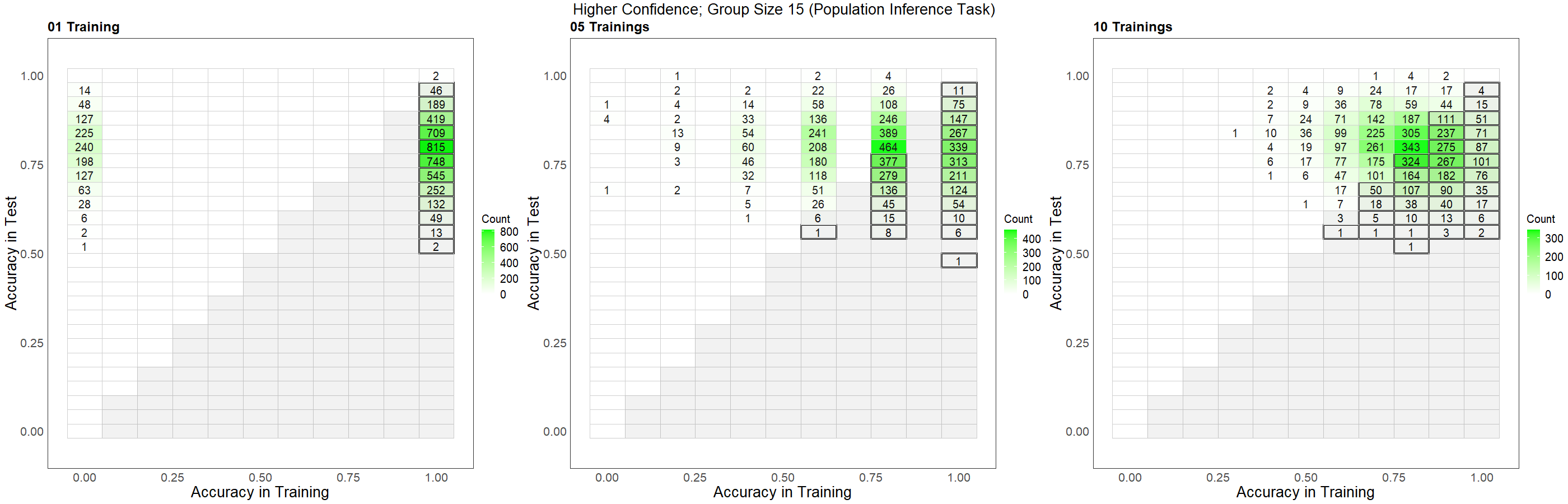


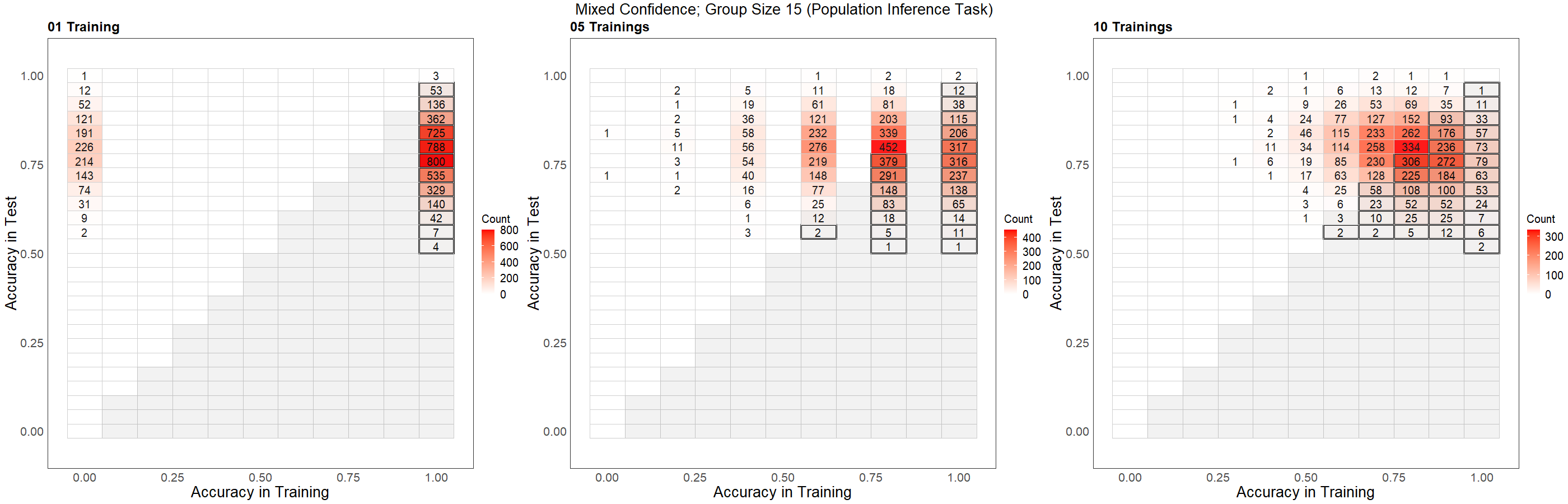


## Relationships comparison task (25 questions); group size 3


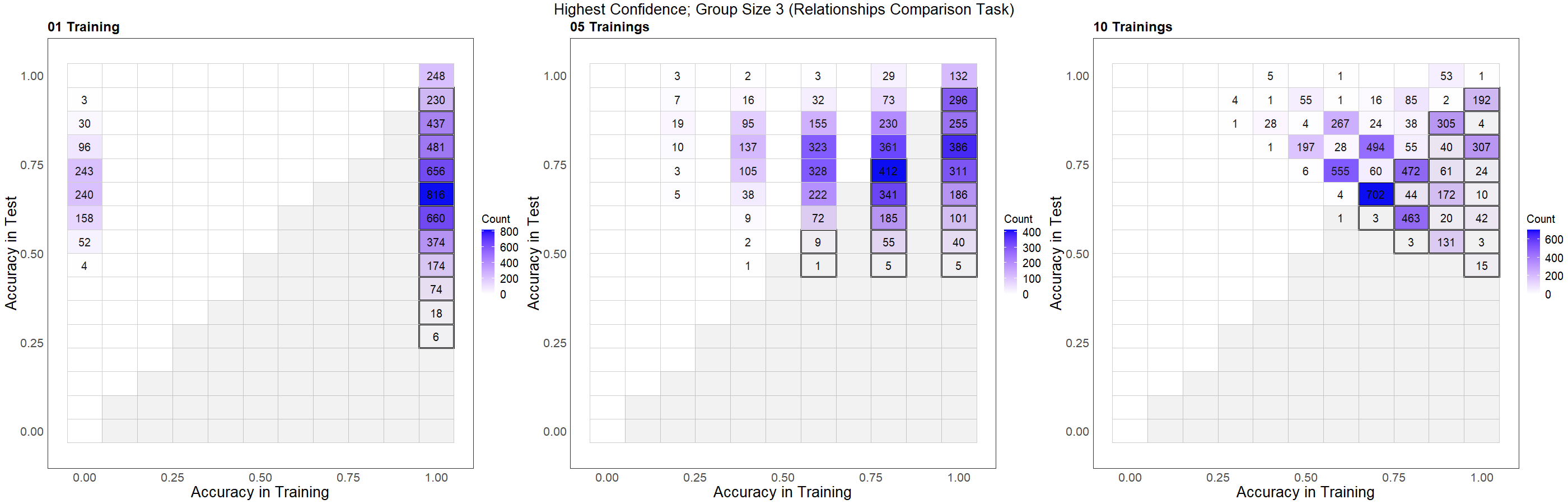


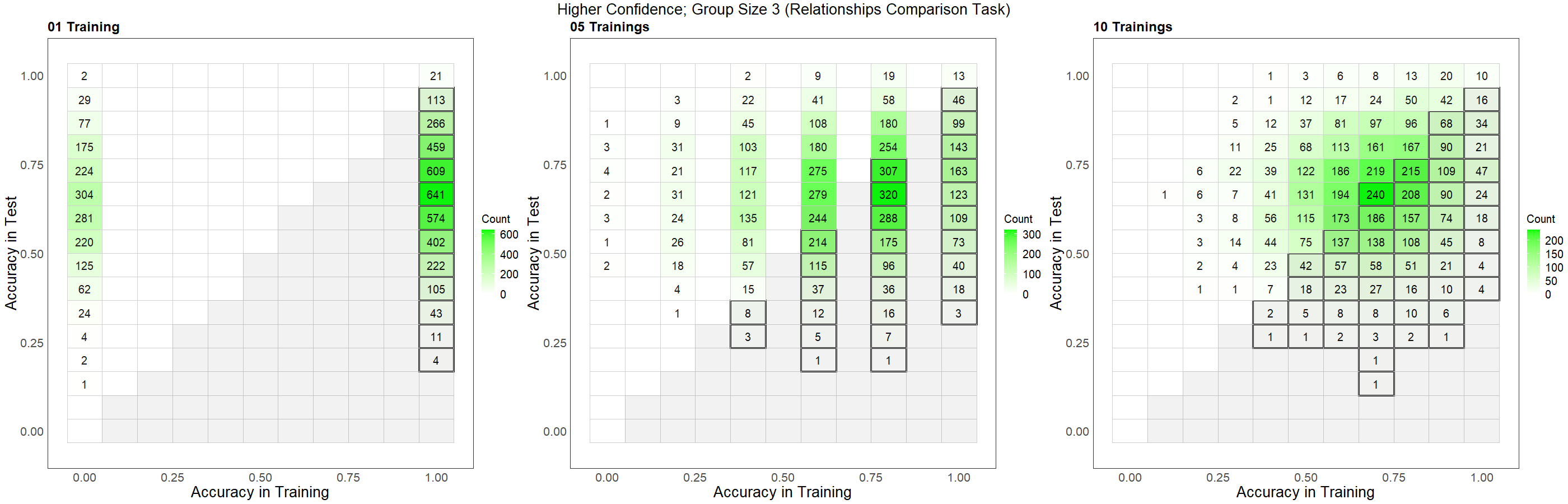


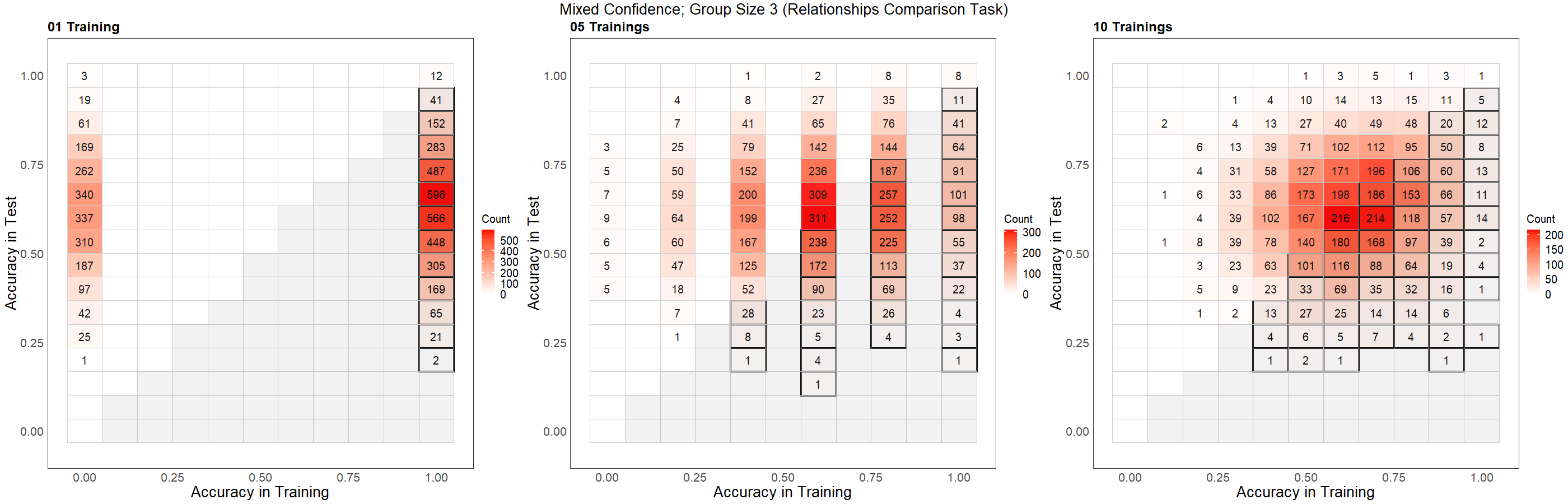


## Relationships comparison task (25 questions); group size 15


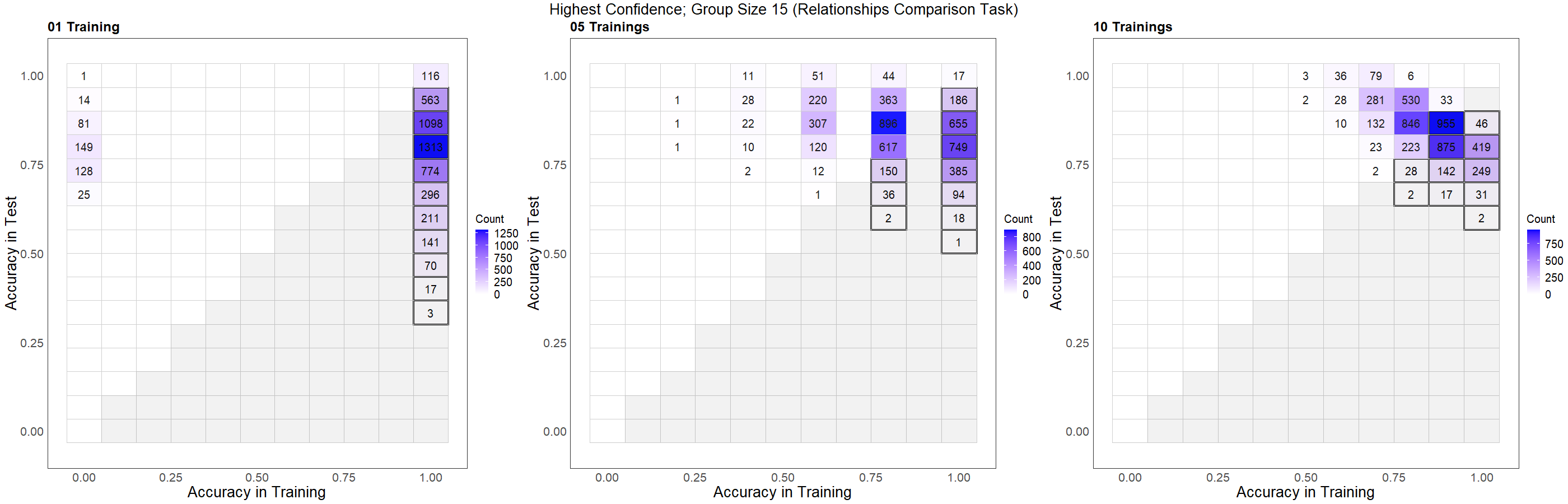


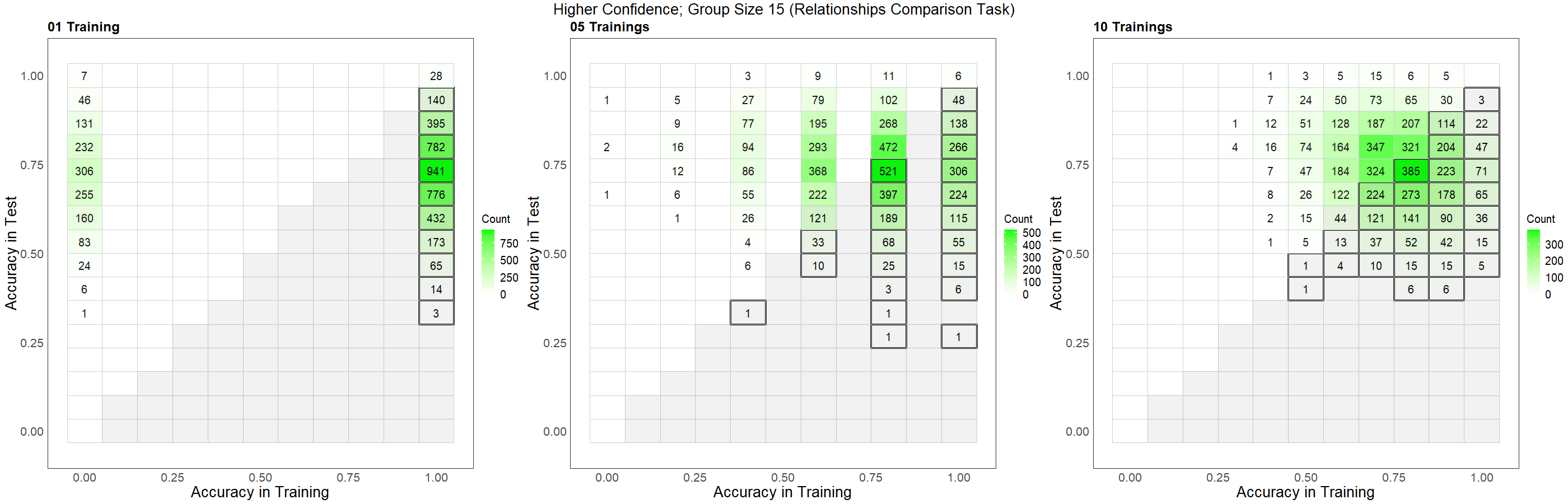


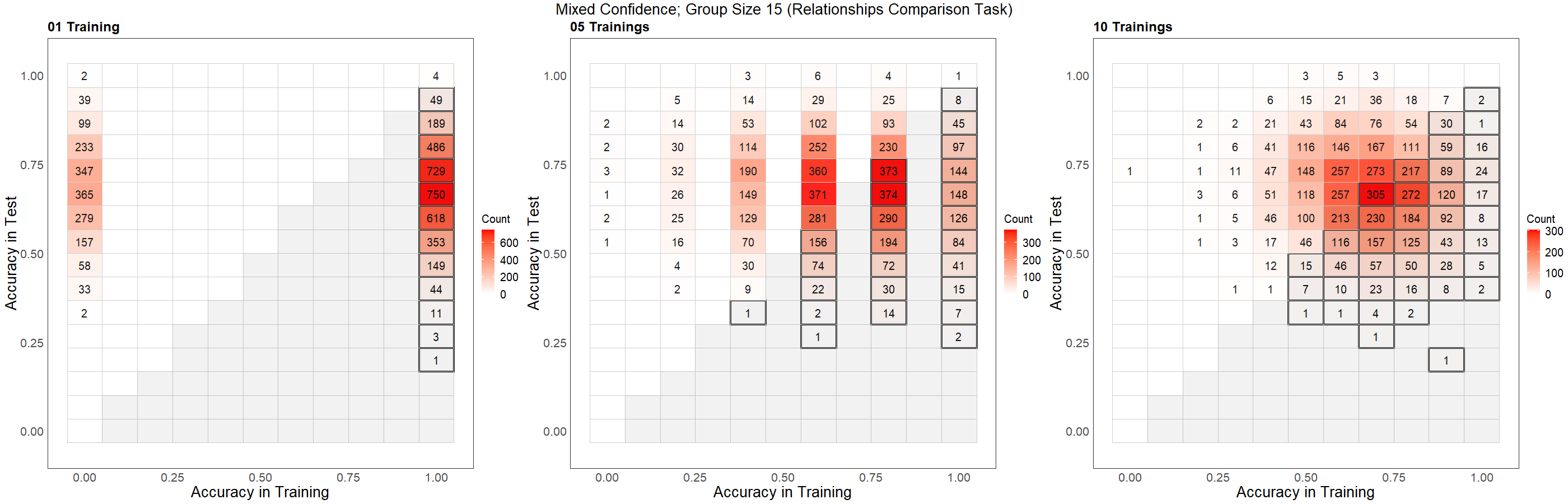


## References

1. Honda H, Matsuka T, Ueda K. Memory-based simple heuristics as attribute substitution: competitive tests of binary choice inference models. Cogn Sci. 2017;41(5):1093-118.

2. Shirasuna M, Honda H, Matsuka T, Ueda K. Familiarity-matching: an ecologically rational heuristic for the relationships-comparison task. Cogn Sci [Internet]. 2020;44(2):e12806. Available from: https://onlinelibrary.wiley.com/doi/full/10.1111/cogs.12806

3. Bürkner PC. Advanced Bayesian multilevel modeling with the R package brms. R J. 2018;10(1):395-411.
