# Supplementary material for: Can individual subjective confidence in training questions predict group performance in test questions?
Source: PLoS One. 2023 Mar 7;18(3):e0280984. doi: 10.1371/journal.pone.0280984 (PMC9990919; doi:10.1371/journal.pone.0280984)
Supplement: Attachment — Submitted filename: Response to Reviewer 3.docx [file pone.0280984.s002.docx]

**Response to Reviewer 3**

We appreciate your careful review of the previously submitted manuscript. We found the comments reasonable and helpful in improving our manuscript. We respect your comments and have attempted to respond to them below.

In the revised manuscript, yellow highlighting indicates the sections with major revisions (e.g., where we have added a new discussion or explanation).

\## Comment

After all, this is not a human prediction task that the authors are evaluating against. The main argument in the paper was stated, "These results suggest that if people aggregate diverse individuals in terms of subjective confidence in prior questions, they will likely avoid decreasing performance in future questions." However, the word "people" here is problematic. This is because, unlike the study in reference [33], in this work they didn't have any human subjects, in an experimental setting, to be given a pool of past decisions and confidence levels from other individuals, and then be asked to make the decision based on them. Instead, what was investigated appears to be simply how a majority voting model can improve its performance in a forward prediction task if taken into account a diverse pool of historical samples rather than a homogeneous one.

**Response**

We appreciate your suggestions. In the initial manuscript, we used the term “people” because "people" gather members and make a group in the real world. As you point out, however, there were no actual human subjects and actual human subjects did not aggregate individuals in the current study because we ran computer simulations.

To reduce this confusion, we avoided the term “people” in describing our simulations of group judgments in the revised manuscript.

\## abstract

These results suggest that when situations are highly uncertain, an effective strategy is to aggregate various individuals regardless of confidence levels in the training questions to avoid decreasing the group accuracy in test questions.

\## P41 L697

As a take home message, individuals’ confidence in training questions can often become a simple and reliable predictor of a group’s test performance but can sometimes become an ill predictor. In highly uncertain situations (e.g., only one training question is available), aggregating only individuals with high confidence in the training question(s) is not always an effective strategy. Rather, aggregating individuals with higher confidence and those with lower confidence in training questions will avoid decreasing group performance in the test questions.

\## Comment

This sentence at line 130 doesn't fully make sense and requires clarifications: "Because some individuals with high confidence may be overconfident or could solve prior question(s) by chance, their errors are likely to be canceled out by mixing individuals with lower confidence into a group (individuals with low confidence may have lack of knowledge and therefore sometimes make random guesses. Thus, their judgments may differ from judgments by individuals with high confidence, which leads to diverse judgments in the group)." It appears that, not matter how confident an individual is, facing a lack of knowledge, they will make random guesses one way or another (either due to over confidence, or due to lack of confidence). If that is the case, wouldn't having a group with either a homogenous or a diverse confidence levels landing the same level of judgement diversity?

**Response**

We appreciate your comments and suggestions. First, as you point out, we agree that the sentence in line 130 was misleading and unclear. We have revised this as follows:

\## P8 L132

Therefore, when a group comprises only individuals with high confidence (called the “high-confidence group”), its training accuracy is unlikely to predict its test accuracy. Even if the accuracy of the high-confidence group is very high in the training question(s), it may decrease in the test questions. On the other hand, if individuals with medium level of confidence are added to a group instead of those with high confidence, the abovementioned “by chance” cases are less likely to occur. Therefore, a group comprising both individuals with high and medium confidence for the training questions (called the “mixed-confidence group”) may be able to better predict test performance based on their training performance. That is, the mixed-confidence group will show a smaller performance gap between test and training accuracy than the high-confidence groups.

\##

Second, as you also point out, not only individuals with low confidence but also those with high confidence sometimes make random guesses. Thus, even in a high-confidence group, individuals’ judgments can sometimes have diversity.

In the revised manuscript, we focused on the frequency of making random guesses when describing the term “random guesses.” That is, individuals with lower confidence will generally have less knowledge than those with high confidence and will more frequently make random guesses. (Thus, more diverse error patterns will more frequently be generated in a mixed-confidence group than in a high-confidence group.)

\## P18 L314

It is expected that patterns of judgments may differ between individuals depending on their levels of confidence. In a certain question, many individuals with high confidence may tend to make similar judgments, whereas those with low confidence may not show such tendencies. This is because individuals with low confidence will generally have less knowledge than those with high confidence and thus will more frequently make random guesses. If so, judgments observed in the mixed-confidence group are more likely to become diverse than those observed in the high-confidence group.

\## Comment

And again, the wording "prior" and "future" can be problematic. On top of the concern with this not about how to predict the decision making behaviors of human individuals (but actually of a much simplified majority voting model), this is not a forward prediction task that the authors are evaluating against, since there is no notion of "time" or "history" here. Spliting a dataset into training and test sets doesn't imply that one has a temporal precedence over the other.

In summary of the past three points, the referee suggests the authors to modify their main arguments and clarify these points, and potentially rephrase their titles and abstracts to correct for controversial terms (e.g. "prior", "future")

**Response**

We appreciate your helpful suggestions. According to your comment, we changed the terms “prior” and “future” to “training” and “test,” respectively (in accordance with a cross validation approach) in the main text, abstract, and title.

\## title

Can individual subjective confidence in “training” questions predict group performance in “test” questions?

\## abstract

In our simulations, we developed a “training-test” approach: We split the questions used in the behavioral experiments into “training questions” (as questions to identify individuals’ confidence levels) and “test questions” (as questions to be solved), similar to the cross-validation method in machine learning.

\## P6 L95

However, in the real world, one must often deal with not only one task set, but also another different task set. In this study, we refer to these two task sets as “training questions” and “test questions,” respectively, in accordance with the cross-validation approach.

\## Comment

Other minor comments:

Fig 6. Please provide legend for different colors in the figure.

**Response**

The colors in Fig 6 do not have special meaning. Thus, we changed the color in the figure to black and white.

Finally, thank you again for your kind and constructive review of our paper.

Masaru Shirasuna, Hidehito Honda
