# Supplementary material for: Can individual subjective confidence in training questions predict group performance in test questions?
Source: PLoS One. 2023 Mar 7;18(3):e0280984. doi: 10.1371/journal.pone.0280984 (PMC9990919; doi:10.1371/journal.pone.0280984)
Supplement: Attachment — Submitted filename: Response to Reviewer2.docx [file pone.0280984.s003.docx]

**Response to Reviewer 2**

We appreciate your careful review of the previously submitted manuscript. We found the comments reasonable and helpful in improving our manuscript. We respect your comments and have attempted to respond to them below.

In the revised manuscript, yellow highlighting indicates the sections with major revisions (e.g., where we have added a new discussion or explanation).

\## Comment

While the previous comments have been addressed well, I would personally like to see a bit more text that relates the research to certain real or hypothetical social phenomena with some more details filled. It can be either in the conclusion part or the limitation section.

**Response**

Thank you for your insightful suggestions. Following your recommendation, we added the following descriptions in the Limitations section.

\## P37 L633

In the real world, there are deceptive or “wicked” environments [32,46] wherein individuals’ confidence and accuracy are not related (i.e., individuals with high confidence sometimes make mistakes). Today, people live in a highly uncertain world. For example, three years ago, no one could predict the outbreak of the COVID-19 pandemic we are currently experiencing; and today, no one can correctly predict the number of infected people three years later. In such uncertain situations, formulating policies based on the judgments of one or few individuals with high confidence to address today’s current problems may not work well for the future’s unknown problems. In fact, our simulations showed that when only one training question was available (i.e., highly uncertain situation), the performance of the highest group largely decreased in the test questions (see Fig 7, especially the blue lines in “01 Training and Group Size 15”). In our study, we did not focus on the effects of task environments, and most of our experimental tasks were non-deceptive or “kind” environments wherein confidence and accuracy are related (as shown in Supporting Information 4). Thus, this issue needs to be examined (e.g., by setting “kind,” “wicked,” and “both” environments and then comparing the group’s judgment performance between these environments). We believe that our arguments will be supported even in a wicked environment. This is because in a wicked environment, aggregating only individuals with high confidence will lead to irrational or inaccurate group judgments, and mixing various individuals in terms of their levels of confidence is more likely to avoid decreasing group performance and may lead to accurate group judgments.

\##

Finally, thank you again for your kind and constructive review of our paper.

Masaru Shirasuna, Hidehito Honda
